# Multimodal Intraoperative Neurophysiological Monitoring in Intramedullary Spinal Cord Tumors: A 10-Year Single Center Experience

**DOI:** 10.3390/cancers16010111

**Published:** 2023-12-25

**Authors:** Maria Pia Tropeano, Zefferino Rossini, Andrea Franzini, Gabriele Capo, Simone Olei, Mario De Robertis, Daniela Milani, Maurizio Fornari, Federico Pessina

**Affiliations:** 1 Department of Biomedical Sciences, Humanitas University, Via Rita Levi Montalcini 4, Pieve Emanuele, 20072 Milan, Italyfederico.pessina@hunimed.eu (F.P.); 2 Neurosurgery Department, IRCCS Humanitas Research Hospital, Via Manzoni 56, Rozzano, 20089 Milan, Italydaniela.milani@humanitas.it (D.M.);

**Keywords:** intramedullary spinal cord tumor, surgery, motor-evoked potentials, D-wave, neurophysiological monitoring

## Abstract

**Simple Summary:**

Despite advances in surgical and imaging techniques, intramedullary spinal cord tumors (IMSCTs) still represent a challenge. Surgical removal of IMCTs carries a substantial risk of spinal cord injury and neurologic morbidity. This study aimed to assess the predictive potential of multimodal intraoperative neurophysiological monitoring (IONM) for functional outcomes in IMSCTs. Clinical data were collected from 64 patients who underwent surgery between 2011 and 2020. Monitoring of motor evoked potentials (MEPs) and somatosensory evoked potentials (SSEPs) was conducted for all patients, while a D-waves recording was obtained in 57 patients. Postoperative neurologic outcomes were measured with Frankel and modified McCormick scales. D-wave monitoring provided valuable insights into motor outcomes, enabling us to proceed with surgery even in cases where MEPs/SSEPs were lost. D-wave monitoring demonstrated superior accuracy and predictive ability compared to MEPs and SSEPs alone. Multimodal IONM has the potential to significantly enhance the extent of tumor resection while minimizing the risk of neurological morbidity in IMSCT surgery.

**Abstract:**

Objective: The study aimed at evaluating the efficacy and the ability of D-wave monitoring combined with somatosensory evoked potentials (SSEPs) and motor evoked potentials (MEPs) to predict functional outcomes in intramedullary spinal cord tumor (IMSCT) surgery. Methods: Between December 2011 and December 2020, all patients harboring IMSCT who underwent surgery at our institution were prospectively collected in a surgical spinal registry and retrospectively analyzed. Patient charts and surgical and histological reports were analyzed. The multimodal IONM included SSEPs, MEPs, and—whenever possible—D-waves. All patients were evaluated using the modified McCormick and Frankel grade at admission and 3, 6, and 12 months of follow-up. Results: Sixty-four patients were enrolled in the study. SSEP and MEP monitoring was performed in all patients. The D-wave was not recordable in seven patients (11%). Significant IONM changes (at least one evoked potential modality) were registered in 26 (41%) of the 64 patients. In five cases (8%) where the SSEPs and MEPs lost and the D-wave permanently dropped by about 50%, patients experienced a permanent deterioration of their neurological status. Multimodal IONM (SSEP, MEP, and D-wave neuromonitoring) significantly predicted postoperative deficits (*p* = 0.0001), with a sensitivity of 100.00% and a specificity of 95.65%. However, D-waves demonstrated significantly higher sensitivity (100%) than MEPs (62.5%) and SSEPs (71.42%) alone. These tests’ specificities were 85.10%, 13.89%, and 17.39%, respectively. Comparing the area under ROC curves (AUCs) of these evoked potentials in 53 patients (where all three modalities of IONM were registered) using the pairwise *t*-test, D-wave monitoring appeared to have higher accuracy and ability to predict postoperative deficits with strong statistical significance compared with MEP and SSEP alone (0.992 vs. 0.798 vs. 0.542; *p* = 0.018 and *p* < 0.001). Conclusion: The use of multimodal IONM showed a statistically significant greater ability to predict postoperative deficits compared with SSEP, MEP, and D-wave monitoring alone. D-wave recording significantly increased the accuracy and clinical value of neurophysiological monitoring in IMSCT tumor resection.

## 1. Introduction

Intramedullary spinal cord tumors (IMSCTs) are a rare clinical entity, accounting for 2–4% of all central nervous system tumors. Among these, spinal cord ependymomas represent the most common subtype in adults [1,2,3,4]. The onset of IMSCTs is often insidious, and these tumors can be symptomatically silent for years [1,2]. Despite the improvement of surgical and imaging techniques, IMSCTs still represent a challenge. Microsurgical resection is the first-line treatment, whereas ablative radiation therapies are considered for residual or recurrent tumors. Nevertheless, the surgical removal of IMSCTs is associated with a significant risk of resection-related spinal cord injury and neurologic dysfunction [2,3,5,6,7,8,9,10].

Intraoperative neurophysiological monitoring (IONM) represents a milestone in the surgical treatment of IMSCTs as a tool to maximize tumor resection and minimize neurological morbidity.

The value of IONM lies in predicting neurological deficits and detecting a neurological injury in time for corrective measures to be taken [3,5].

Several surgical series about IMSCTs have been published in the last two decades. However, due to the rarity of the disease, these series are often small, span a long time, and lack homogeneity in the application of monitoring. Moreover, the role of IONM in preventing, rather than merely predicting, motor deficits is still a matter of debate [3,4,5,9,10,11,12,13,14].

This study reported our experience in the surgical management of IMSCTs. All procedures were performed by a single spine surgeon (M.F) with the same standardized IONM protocol provided by a single neurophysiologist (D.M.)

Multimodal IONM includes somatosensory evoked potentials (SSEPs), motor evoked potentials (MEPs), D-waves, and electromyography.

The aims of this study were to evaluate the ability of multimodal IONM to predict new postoperative neurological deficits after IMSCT resection and statistically compare the accuracy of SSEPs, MEPs, and D-waves using receiver operating characteristic (ROC) curves.

## 2. Methods

### 2.1. Patient Population

Between December 2011 and December 2020, all patients who underwent surgery for IMSCTs at our institution were prospectively collected in a surgical spinal registry and retrospectively analyzed. Patient charts and surgical and histological reports were analyzed. Informed consent to archive and process patients’ data in an anonymous form was also obtained. Permission from our institutional ethics committee was obtained for this study (N° 48/23).

### 2.2. Anesthesia

The anesthetic protocol for procedures with IONM at our institution includes orotracheal intubation and continuous infusion of Propofol (100–150 mg/kg/min) and Fentanil (1 mg/kg/h) without halogenated anesthetics. Bolus injections are avoided.

### 2.3. Multimodal Intraoperative Neurophysiological Monitoring

IONM was used in all cases. Somatosensory evoked potentials (SSEPs) and motor evoked potentials (MEPs and D-wave) monitoring were attempted in every patient. A summary of our multimodal IONM protocol has been described in Table 1.

### 2.4. Data Collection

Relevant clinical data were obtained from clinical notes, operative notes, and telephone conversations with the patients or relatives. Functional and neurological outcomes were assessed with the Frankel [15] and modified McCormick (MMS) [16] scales (Table 2 and Table 3). Neurological examination was performed immediately following microsurgical resection, three and six months postoperatively, and yearly after that. The extent of surgical resection was assessed on the magnetic resonance images (MRI) acquired within a week after surgery and graded according to the criteria listed in Table 4. Tumor recurrences were detected with a follow-up MRI examination, which was acquired six months following surgery and every six months after that. Pathological examination confirmed histopathological diagnosis.

### 2.5. Statistical Analysis

The following variables were assessed: sensitivity, specificity, positive predictive value (PPV), and negative predictive value (NPV). Sensitivity was defined as the probability that IONM would identify newly developed true neurophysiological deficits. Specificity was defined as the probability that IONM would correctly identify no significant neurophysiological deficit. The PPV was defined as the probability that a significant IONM change reflected a true neurophysiological deficit. In contrast, the NPV was the probability that a finding of no IONM change truly reflected no significant neurophysiological deficit.

Each value was assessed as a transitory deficit (24 h after surgery) and a sustained deficit at follow-up (six months after surgery).

Receiver operating characteristic (ROC) curves were used to compare intraoperative evoked potential changes to the onset of new persistent motor post-operative deficit. A Pairwise *t*-test was used to compare the SEPs, MEPs, and D-Wave ROC curves.

Survival curves and mean survival time for recurrence were estimated using the Kaplan–Meier method. The log-rank test was used to estimate the overall significance of each variable included in the analysis. Using the Cox proportional hazards regressions, hazard ratios (HR) and 95% confidence intervals (CI) were obtained. The Cox regression model was also used to study the effects of multiple covariates on patients’ survival.

Analyses were performed with SPSS 21.0 (SPSS Inc. Chicago, IL, USA). A statistical significance was set at *p* < 0.05.

## 3. Results

We collected data for 64 consecutive patients. Clinical, radiographic, and operative variables are shown in Table 5. At the time of surgery, the mean age was 48.12 ± 14.55 years (range 17–79 years). Thirty-four patients presented with Frankel Grade D symptoms (53%) and mild/moderate motor or sensory deficit (MMS grades II–III; 56%). The median preoperative MMS score was 2. Before surgical resection, the mean duration of symptoms was 16.89 months (range 1–108 months). Fifty-three patients (83%) presented with sensory disturbances, 30 (47%) with pain, 31 (48%) with motor weakness and gait ataxia, and 15 (23%) with sphincteric dysfunction. Almost all patients showed more than one symptom at the diagnosis.

Pathological diagnosis was made in all cases. Ependymomas were the most frequent tumor subtype (38 pts, 59%), followed by cavernous angioma (6 pts, 9%) and hemangioblastomas (6 pts, 9%), WHO Grade I/II astrocytomas (7 pts, 11%), and intramedullary spinal metastases (3 pts, 5%) (Table 6). Among patients with hemangioblastomas, 30% (2 of 6) had a known von Hippel-Lindau (VHL) syndrome. Other pathological types included a solitary fibrous tumor (1 pt, 2%), a mesenchymal tumor (2 pts, 3%), and a paraganglioma (1 pt, 2%). The most common location of IMSCT was the cervical spine.

### 3.1. Surgical Data

The mean operative time was 231.65 ± 60.98 min (range 135–503). Gross total tumor resection (GTR) was achieved in 48 patients (75%), subtotal tumor resection (STR) in 12 patients (19%), and biopsy was performed in 4 patients (6%) (Table 7). As seen on pre-operative MRI, syringomyelia predicted GTR rates (54% of patients with preoperative syringomyelia underwent GTR versus 39% of patients without syringomyelia undergoing GTR, *p* = 0.0017).

### 3.2. Monitorability of IONM

SSEP and MEP monitoring were performed in all patients. Recording of D-waves was achieved in 57 out of 64 patients. The D-wave was not monitorable in 7 patients (11%) with severe neurological deficits before surgery (modified MMS IV) and lower thoracic lesions.

### 3.3. Intraoperative IONM Changes

In 26 (41%) of 64 patients registered significant IONM changes (at least one evoked potential modality). In four cases (6%), these events resolved after a brief stop of surgery, allowing a gross total resection to be achieved (stop-and-go surgery). We observed the persistent loss of at least one of the three evoked potentials in 22 of the remaining patients (34%). Five patients (8%) experienced a permanent worsening of their neurological status. In these cases, SSEPs and MEPs were lost, and the D-wave permanently dropped by about 50%. In 2 cases, the procedure was definitely stopped (halt surgery) to prevent permanent paraplegia after numerous attempts to restart it with a different approach. A residual tumor was left in place. These patients showed a persistent new motor deficit at the 6-month follow-up. In 17 patients (30%), the D-wave remained stable or decreased no more than 50% in combination with changes in MEPs and/or SSEPs, allowing us to continue the surgical procedure. Among these patients, eight experienced a new transitory motor deficit, while the other nine patients had no new postoperative deficit. In the remaining 44 (77%) patients, the combined use of SSEP, MEP, and D-wave IONM predicted a good neurological outcome or no new deficits. There was no relationship between the time of surgery and the occurrence of significant changes in IONM or loss of accuracy.

#### 3.3.1. D-Wave

None of the 48 patients who had a recordable and stable D-wave showed a new permanent motor deficit at follow-up (true negative) (Table 8). Indeed, in these patients, D-wave monitoring gave us significant information about the motor outcomes and allowed us to proceed with surgery even when there was MEP/SSEP loss. In 17 (30%) of 57 patients, the D-wave was stable or decreased by no more than 50% in combination with deterioration of MEPs and/or SSEPs. In these cases, we continue surgery after a temporary stop. In 5 (9%) patients, the D-wave permanently decreased by approximately 50% with concomitant complete loss of low SSEPs and MEPs. All five patients experienced a worsening in the neurological outcome without recovery at 6-month follow-up.

In three patients, the D-wave permanently decreased by approximately 50% with concomitant complete loss of MEPs but without loss of SSEPs. All three patients emerged from surgery without new neurological deficits, and we consider the results of D-wave registrations to be false positives.

#### 3.3.2. SSEP

SSEPs could be recorded in 64 patients. Among these, 42 patients (65%) showed stable SSEP recordings during surgery. Four (9%) patients emerged from surgery with a new functional deficit in the immediate postoperative stage (false negative). Two patients returned to their preoperative status during hospitalization; one patient experienced resolution before the 6-month follow-up, while one patient displayed a permanent neurological deficit.

Of 18 patients who had permanent SSEP loss, 10 showed a functional deficit (true positive) in the early postoperative stage. Among these, five patients developed deficits resolved at the last follow-up (new transitory deficit), while five patients presented the same deficits at the 6-month follow-up (new permanent deficit). Indeed, these patients showed permanent MEP and D-wave loss. The other eight patients who experienced permanent SSEP changes during surgery had no postoperative deficit (false positive).

#### 3.3.3. MEP

Overall, 10 of 40 patients with stable MEP during surgery showed new deficits in the immediate postoperative stage (false negative), which improved during the hospital stay, while 33 experienced no new neurological deficit (true negative). Of 20 patients with significant MEP changes, 5 had no postoperative deficits (false positive), 5 showed an improvement in neurological status, and 10 patients developed a new motor deficit after surgery. Five of these patients also showed a D-wave loss.

### 3.4. Sensitivity, Specificity, and ROC Curves

Sensitivity, specificity, positive predictive value (PPV), negative predictive value (NPV), positive likelihood ratio, and negative likelihood ratio analyses of each evoked potential are shown in Table 9.

Multimodal IONM (SSEPs, MEPs, and D-wave) significantly predicted the onset of new postoperative deficits (*p* = 0.0001) with a sensitivity of 100.00% and a specificity of 95.65%.

However, the sensitivity of D-waves was significantly higher (100%) than MEPs (62.5%) and SSEPs (71.42%). SSEP and MEP showed a specificity of 85.10%, 13.89%, and 17.39%, respectively.

The pairwise *t*-test was used in order to compare the area under ROC curves (AUCs) of these intraoperative neurophysiological modalities.

In 53 patients harboring IMSCTs, all three modalities of IONM were recorded.

D-wave monitoring appeared to have higher accuracy and ability to predict postoperative deficits with strong statistical significance compared with MEPs and SSEPs alone (0.992 vs. 0.798 vs. 0.542; *p* = 0.018 and *p* < 0.001, Table 9).

**Figure 1:**
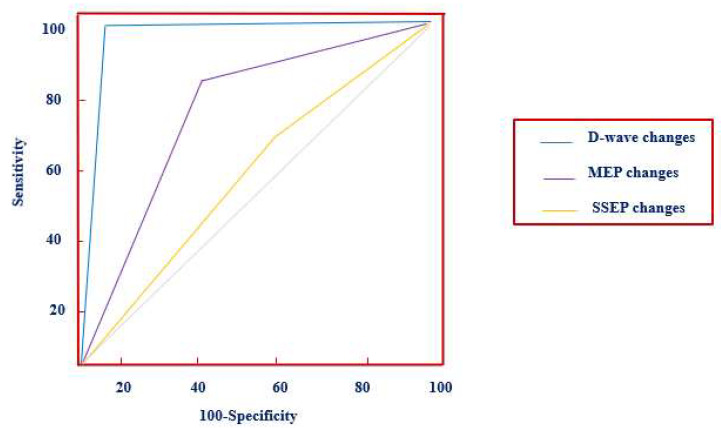
ROC curves for the D-wave, MEP, and SSEP.

In Figure 1, the ROC curves are shown.

### 3.5. Factors-Related IONM Changes

At univariate analysis, age older than 65 years (*p* = 0.03) and astrocytoma histology (*p* = 0.001) are significantly associated with a high probability of IONM changes during surgery. On multiple logistic regression, the only independent risk factor associated with significant IONM changes was astrocytoma histology (*p* = 0.0027).

These results are summarized in Table 10.

## 4. Discussion

Despite the recent improvements in the management of IMSCTs, surgery still carries a significant risk of intraoperative damage, with morbidity ranging from 3.7% to 7.5% [4,17,18,19,20]. IONM represents the most effective tool for identifying and monitoring the functional integrity of both the spinal cord and the nerve roots in real time [21,22,23,24,25,26,27,28,29,30].

As mentioned, the concept behind IONM is to guide the surgeons and allow them to prevent injury to the spinal cord through the production of signal changes rather than merely predict.

### 4.1. Monitorability, Accuracy, and Clinical Value of IONM

The value of IONM in IMSCT surgery has largely been investigated, although their role has been questioned in the recent guidelines on the use of IONMs in spine surgery published by Hadley et al. [7].

The use of IONM is not advised by the Authors as a therapeutic tool during IMSCT surgery (level II) or other spinal cord/spinal column surgery (level III). The authors only recommended the use of IONM as a diagnostic tool to assess spinal cord integrity during spine procedures (level I).

In our experience, multimodal IONM is a valuable tool for IMSCT surgery.

Combining monitoring modalities could give a more precise perspective on intraoperative morbidity and functional outcomes. Indeed, each IONM modality has technical, clinical, and predictive value limitations when used alone. The accuracy of multimodal IONM, which combines D-wave with MEP and SSEP monitoring, was very high, with sensitivity and specificity of 100% and 95.6%, respectively, and having a PPV and NPV of 77.7% and 100%, respectively.

In our study, the D-wave monitoring showed higher accuracy and ability to predict postoperative deficits with strong statistical significance than MEP and SSEP alone (0.992 vs. 0.798 vs. 0.542; *p* = 0.018 and *p* < 0.001, Table 9) comparing the AUC of each evoked potential. Based on these findings, the D-wave recording considerably improved the accuracy of neurophysiological monitoring during IMSCT surgery.

The recording of the D-wave in IMSCT surgery is a critical issue. Preexisting impairments or poor preoperative neurological status are highly correlated with the baseline ability to detect the D-waves. In our series, the D-wave was not monitorable at the beginning of surgery in 7 patients (11%) because of severe neurological impairment. This data is slightly lower than the present literature, which reported that the D-wave is not recordable in about 30% of spinal cord surgery [8]. This finding supported the concept that patients with severe neurological deficits who underwent surgery had a lower monitorability rate than those with normal neurological function. In these patients, the clinical value of IONM in preventing neurological deficits is limited.

### 4.2. Limits of the Study

Our study was not without limitations. This was a single-center prospective cohort study without a non-IONM group for comparison, which prevented further regression analysis from being conducted. In addition, the sample size is relatively small because of the rarity of the studied condition. This group of patients was heterogeneous and included a diverse age range. We stopped our follow-up at 24 months post-surgery. It is possible that an extended follow-up period would be required to detect later changes. However, monitoring was performed by the same team throughout the study (D.M.), and all operations were performed by a single spine surgeon (MF), thus limiting a possible source of bias.

## 5. Conclusions

In our study, the use of combined multimodal IONM showed a greater statistically significant ability to predict postoperative deficits compared with SSEP, MEP, and D-wave monitoring alone.

D-wave recording significantly increased the accuracy and clinical value of neurophysiological monitoring in IMSCT resection.

Although still a matter of debate in the literature, multimodal IONM may be greatly helpful in maximizing tumor resection and minimizing neurological morbidity in IMSCT surgery.

## Figures and Tables

**Table 1:** Multimodal IONM.

Parameter	Stimulation	Registration	Alarm Criteria
SSEPs	median nerve at the wrist posterior tibial nerve at the ankle intensity 40 mA, duration 0.2 ms, repetition rate 4.3 Hz	CZ′-FZ: legs C3′/C4′-FZ: arms	50% drop in amplitude 10% prolongation in latency
MEPs	TES with the multipulse technique Short trains of 5–7 square-wave stimuli duration 0.5 ms interstimulus interval (ISI) of 4 ms frequency of up to 2 Hz C1/C2: right arm C2/C1: left arm Cz-Fz: legs intensity < 200 mA.	Arm: abductor pollicis brevis (APB) extensor digitorum communis (EDC) Leg: tibialis anterior (TA) abductor hallucis (ABH)	Amplitude modification
D-Wave	single transcranial pulse of 0.5 ms duration same TES electrodes as for the MEPs	epidural electrode caudal to the lesion	decrease >50% of the baseline amplitude

**Table 2:** Frankel grading scale.

Grade	Description
A	Complete motor and sensory loss
B	Complete motor loss, incomplete sensory loss
C	Incomplete motor loss without practical use
D	Incomplete motor loss, able to ambulate with or without aids
E	Normal motor and sensory function

**Table 3:** Modified McCormick grading scale.

Score	Description
I	Intact neurologically; normal ambulation; minimal dysesthesia
II	Mild motor or sensory deficit; functional independence
III	Moderate deficit; limitation of function; independent w/ external aid
IV	Severe motor or sensory deficit; limited function; dependent
V	Paraplegia or quadriplegia, even w/ flickering movement

**Table 4:** Extent of resection.

GTR	no tumor remnant detectable at the end of surgery or on postoperative MRI
STR	complete resection of the tumor mass with a small remnant detectable on the postoperative MRI
biopsy	less than 50% reduction in the tumor mass

**Table 5:** Demographic, clinical, and radiological characteristics.

Clinical and Radiological Variables	No of Patients/Value (%)
Age (years)	
Mean	48.3 ± 14.55
Range	17–79
Sex	
Male	35
Female	31
Symptoms at presentation	
Pain	30
Motor weakness	31
Sensory disturbance	53
Gait ataxia	31
Sphinteric function	15
Duration of symptoms (mo)	
Mean	16.89
Range	1–108
Preoperative Mc Cormick	
I	21
II	23
III	13
IV	7
V	0
Preoperative Frankel Grade	
A	0
B	2
C	6
D	34
E	22
Location	
Cervical	21
Cervico-thoracic	9
Thoracic + conus	34
No. of spine segments involved	
Mean	2.58 ± 1.30
Range	1–6

**Table 6:** Histological features of our series.

Histology	
Ependymoma	
Myxopapillary ependymoma	2
WHO Grade II	35
WHO Grade III	1
Astrocytoma	
WHO Grade I/II	4
WHO Grade III/IV	3
Hemangioblastoma	6
Cavernous angioma	6
Metastatic lesions	3
Miscellaneous	4

**Table 7:** Surgical data.

Surgical Data	
Duration of hospitalization in days	
Mean ± SD	14.25 ± 10.15
Range	5–56
Extent of tumor resection	
Gross total resection	48
Subtotal resection	12
Biopsy	4
Operative time (min)	
Mean ± SD	231.65 ± 60.98
Range	135–503

**Table 8:** Sensitivity, specificity, positive predictive value (PPV), negative predictive value (NPV), likelihood ratio positive (LH+), likelihood ratio negative (LH−), area under the curve (AUC), and statistical analysis of IONM.

	Multimodal IONM (SSEP + MEP + D-Wave)	SSEP	MEP	D-Wave
True negative	44	40	33	45
True positive	7	10	16	7
False negative	0	6	10	0
False positive	2	8	5	5
Sensitivity	100.00%	71.42%	62.50%	100.00%
Specificity	95.65%	17.39%	13.89%	85.10%
Positive predictive value	77.75%	55.55%	75.00%	60.00%
Negative predictive value	100%	90.47%	77.50%	100%
Likelihood ratio +	23	0.86	0.725	6.67
Likelihood ratio −	0	1.64	2.69	0
AUC	0.978	0.542	0.798	0.992
95%CI	0.894–0.997	0.399–0.68	0.5820–0.836	0.878–0.996
*p* Value	**0.0001**	0.71	0.002	**0.0001**

**Table 9:** Comparison between D-Wave, SSEP, and MEP ROC curves.

	Value
D-wave vs. SSEP	
D-wave AUC	0.992
SSEP AUC	0.542
95% CI	0.357–0.901
*p* value	0.0018
D-wave vs. MEP	
D-wave AUC	0.992
MEP AUC	0.798
95% CI	0.048–0.908
*p* value	<0.001

**Table 10:** Univariate and multivariate analysis of factors associated with a high probability of IONM changes during surgery.

	Univariate Analysis			Multivariate Analysis		
Predictor	Hazard Ratio	95% CI	*p* Value	Hazard Ratio	95% CI	*p* Value
Age > 65	0.99	0.95–1.02	**0.03**	0.98	0.85–1.05	**0.001**
Sex ^1^	0.6	0.2–1.6	0.85	0.89	0.1–1.9	0.75
Histology *			0.001			0.001
Astrocitoma	9.1	2.36–35.6	**0.001**	8.01	1.44–42.35	**0.0027**
Hemangioblastoma	1.6	0.18–13.71	0.62	3.2	0.5–19.8	0.1
Others	1.5	0.29–7.8	0.66	0.24	0.14–4.2	0.33
Preop Frankel Grade	0.45	0.17–1.20	0.11	0.72	0.51–4.79	0.44
Preop McCormick score	1.56	0.75–2.66	0.86	1.03	0.37–1.38	0.32
Region of spinal cord ∫			0.32			0.41
Thoracic	2.5	0.75–8.48	0.13	2.5	0.58–12.2	0.24
Conus medullaris	4.5	0.49–41.3	0.17	35.13	1.09–11.32	0.51
No, of spine segments involved	0.7	0.65–1.23	0.58	0.67	0.44–1.005	0.053

^1^ reference = male; * reference = ependymoma; ∫ reference = cervical.

## Data Availability

Data are contained within the article.
